# Exploring awareness of obstetric fistula in Eastern and Northern Nigeria: perceived causes, symptoms, and availability of treatment services

**DOI:** 10.1186/s41256-022-00264-0

**Published:** 2022-08-18

**Authors:** Emmanuel Kelechi Nwala, Charles Nwaigwe, Pooja Sripad, Charlotte E. Warren, Salisu Ishaku, Solomon Kongyamba

**Affiliations:** 1 Maternal, Newborn and Child Health, Population Council, Nigeria, House 4, No 16 Mafemi Crescent, Off Solomon Lar Way, Utako, Abuja, Nigeria; 2 Population Council, Washington, DC USA

**Keywords:** Women with obstetric fistula, Fistula awareness, Knowledge about fistula, Treatment seeking behavior, Fistula repair services

## Abstract

**Background:**

Evidence suggests that there are approximately two female genital fistula cases per 1000 women of reproductive age in sub-Saharan Africa. It is estimated that more than 200,000 women are affected by fistula in Nigeria, primarily due to obstetric causes. Awareness has been indicated as a risk factor for the development of obstetric fistula. This study explored the awareness of obstetric fistula such as causes, symptoms, and availability of and access to treatment services in southeastern and northwestern Nigeria.

**Methods:**

An exploratory qualitative study design was used to conduct this research in Kano and Ebonyi states in northern and eastern Nigeria, respectively. A total of six (6) focus group discussions were conducted with three categories of participants: women who were successfully repaired and discharged (n = 2), community-married men (n = 2), and women (n = 2). Forty-four (44) In-depth interviews were conducted three categories of participants: 18 with women with fistula who were either awaiting repair or had been repaired; 6 family member caregivers accompanying fistula patients, and 20 health service providers at fistula repair centers. We developed a data analysis plan based on the emerging themes. The transcripts and field notes were imported into QSR Nvivo version 11 and coded accordingly. Content and thematic analysis was run by inductively drawing themes based on the elicited information from participants.

**Results:**

There was lack of knowledge of obstetric fistula and its causes among married men and women in the community, caregivers, and some patients were unaware of what caused their fistula for years. In this study, none of the community men and women nor caregivers correctly identified the causes and symptoms of a fistula or knew where to seek treatment. Knowledge about fistula was more common among women who had undergone repairs. Some repaired women attributed the cause of fistula to the providers who attended to them during delivery.

**Conclusions:**

Findings reveal a widespread lack of awareness of obstetric fistula onset and awareness of the availability of repair services at the community level. There is an urgent need to explore multi-pronged strategies for increasing awareness of obstetric fistula and available treatment services among women and other community members.

## Background

The hardship suffered by a woman living with genital fistula, usually obstetric in origin, has attracted worldwide attention. Despite the global campaign against fistula as a debilitating and traumatic condition, awareness within affected countries is still low [1, 2]. Evidence suggests that sub-Saharan Africa (SSA) has a fistula prevalence of approximately 2 per 1000 women of reproductive age and it is estimated that, in Nigeria more than 200,000 women are affected by obstetric fistula [2]. Reports from other parts of Africa indicate lack of knowledge of fistula especially among community men and women and that lack of knowledge of available services may lead to non-use of services or inappropriate health-seeking behavior [3]. Studies in health-seeking behavior have further demonstrated the likelihood that knowledge translates into practice [4]. The decision to seek or not to seek care may be compounded by other factors including lack of awareness of signs and symptoms, transportation, and opportunity costs including travel cost and time lost, and provider behaviors and limited facility resources [3, 5, 6].

Awareness has been indicated as a risk factor for the development of obstetric fistula. For example, short mothers understand that they are at high risk of pregnancy, there is a tendency for increased demand for quality emergency obstetric services [7]. A prevailing lack of awareness and knowledge of fistula, its causes, consequences, and care options affect women’s ability to seek care. Knowledge of fistula is low across rural and urban settings [8]. Studies have highlighted other factors which cause delays in seeking care and may be linked to poverty status [9, 10]. Evaluation of perceptions of fistula among rural dwellers in SSA shows that prolonged labor, mistakes by doctors/nurses while delivering by cesarean section, age of the mother, and health system factors comprise the reported causes of fistula [9, 11, 12]. Other studies in SSA have reported lack of knowledge of fistula causes and symptoms [3, 13]. Though there is recognition that fistula is a concern in Nigeria limited peer-reviewed evidence exists looking deeply at the awareness barrier concerning causes of and repair options for fistula. The surgical closure of an obstetric fistula is reportedly the most effective way to treat or repair obstetric fistula [14]. Success rates are high and measures up to 80–97%. Surgeons most times wait for three months after a delivery to repair, allowing time for spontaneous closure and for granulation tissue to disappear [15]. In Nigeria, despite the established National Obstetric Fistula Centres (NOFICs) across the six geo-political zones and the establishment of free fistula care in 2015 [16], many affected women remain unrepaired. In southeastern Nigeria, while both men and women perceive fistula to be a punishment from the gods, gendered views persist; men blame the woman for unforeseen natural forces or destiny and women blame the traditional birth attendants and other providers who delivered them [17]. Regional efforts to increase community awareness of fistula over time include the use of mobile phones for fistula screening in Kenya and Tanzania [1], community-based screening in rural Nigeria, and voluntary community sensitization and village safe motherhood initiatives in rural Guinea [10]. No peer-reviewed studies in Nigeria have described in-depth the knowledge of where to access fistula care among community men, women and caregivers in eastern and northern Nigeria. To fill this gap, the purpose of this study was to explore the awareness of fistula causes, symptoms, and the availability of and accessibility to treatment services in southeastern and northwestern Nigeria.

## Methods

### Study area

This study was conducted in the Ebonyi and Kano states of Nigeria. Ebonyi state usually branded "Salt of the Nation", is in the southeastern part of the country and is comprised of 13 local government areas (LGAs) with the main source of income predominantly from farming. Public sector facilities comprise 370 (66.8%) of the total facilities while 184 (33.2%) are private. A strong presence of six mission hospitals provides about 60 percent of health services in the state [17]. Kano state, located in the northwestern geopolitical zone of Nigeria, is popularly known as a center for commerce, industry, and agriculture with millet, beans, sorghum, and maize being produced in large quantities [18]. Health services in the state are more concentrated in the urban areas. Eighty-nine percent (89%) of all doctors and 73% of nurses in the employment of state government are in the metropolis of Kano town. Kano state has 1030 public-owned primary health care (PHC) facilities, out of which 58% are health posts and 42% health centers that provide mostly preventive services with little or no clinical care [18]. Health indices demonstrate marked differences among Ebonyi and Kano states regarding receipt of antenatal care, skilled and facility delivery, fertility, and family size preferences [19]. The proportion of women receiving skilled ANC varied (85% in Ebonyi and 64% in Kano. Skilled delivery is lower in Kano (14%) compared to Ebonyi (62%), while the median age at first birth was 21.4 years in Ebonyi and 18.2 years in Kano [19].

### Study design

An exploratory qualitative study design consisting of six (6) focus group discussions (FGDs) and forty-four (44) in-depth interviews (IDIs) was conducted with a range of participants in two urban and two rural LGAs in Kano and Ebonyi in northwestern and southeastern Nigeria, respectively. Despite FGDs, IDIs were selected to get in-dept and confidential understanding of the perceived and actual experiences of women with fistula, experiences of family members or caregivers as well as health providers in dealing with women with fistula. Also, IDIs yielded robust and confidential data given the sensitivity of the subject matter to compliment the FGDs.

The FGDs were conducted with three categories of participants (women with fistula, community women, and community men). A total of six (6) FGDs were conducted: women who were successfully repaired and discharged (n = 2, one in each state at the NOFIC), community married men (n = 2), and community married women (n = 2) in each of the states. The FGDs provided opportunity to discuss mixed views and to share both indirect experiences within the neighborhood.

The IDIs were conducted with three categories of participants (18 women with fistula who were either awaiting repair or had been repaired, 6 caregivers accompanying fistula patients for repair, and 20 health service providers (surgeons, matrons, nurses, and health counselors) working at the two NOFICs (Kano and Ebonyi). The caregivers consisted of spouses and female relative (daughter, sister or mother) who accompanied the women to the NOFIC (fistula center). All women aged 15–49 years who had fistula were included in the both FGD and IDIs. The study included married men and women aged 15–55 years given their exposure to childbirth, interactions with other married peers in their locality, and shared pregnancy, labor, and delivery experiences.

### Selection of participants and data collection

For the FGDs, the research team collaborated with the safe motherhood coordinators of each state to identify opinion leaders and community mobilizers who facilitated FGD recruitment. Eight to ten (8–10) participants who met the inclusion criteria were asked to join the discussion on a specific date and time at an agreed and convenient venue. FGDs were conducted by a trained facilitator and note-taker. During the discussion, community members were asked about women's prevailing health problems, problems faced by women during childbirth, knowledge, and perception around fistula, causes of fistula, whether participants have seen women who suffer fistula, health-seeking behavior of people with fistula, barriers to seeking fistula care and possible ways to promote awareness of fistula in the community.

For the IDIs, a selected social worker at each NOFIC identified women awaiting repair or post-repair discharge as potential respondents. Additionally, caregivers who accompanied the women to the repair centers were identified and also interviewed. Women and caregivers were asked about their awareness about fistula, its causes, and influencing actors to seek care, how patients get to know about any fistula center, perceptions about the treatment and the NOFIC itself, the women’s relationship with spouse and other relatives. At NOFICs, women are managed by a range of professionals who are obstetrics and gynecology specialists. Providers at the fistula centers were interviewed to understand their views about treating women with fistula. The social worker also recruited providers at the NOFICs to be interviewed about their roles in the fistula centers, and their perceptions regarding barriers to and enablers of accessing fistula care. Interview guides were developed and pretested for language and relevance to guide the facilitators during the discussions. Female research assistants were recruited to interview female participants (women with fistula, caregivers, and female health providers at the NOFICs), while male research assistants interviewed the community men and male health providers. The interviews and discussions were conducted in the local language (Hausa and Igbo), recorded, transcribed verbatim, and translated back to English for analysis purposes. To ensure the translation's validity and accuracy, language consultants (Hausa and Igbo) were engaged for the transcription. In addition, the research team, comprising of English and local language speakers (Hausa and Igbo) reviewed the transcripts and compared both Hausa, Igbo and English versions to ensure that the original information was not lost or misinterpreted.

### Data processing and analysis

After the FGD and IDI interviews, audio files and transcripts were assigned unique identifiers to ensure participant privacy and enable referencing. Transcripts and field notes were carefully read for initial content analysis to identify emerging themes or concepts which later represented the nodes. The data analysis plan and coding tree were drafted inductively based on the emerging themes. The transcripts and field notes were imported into QSR Nvivo version 11 [20] and coded accordingly into the nodes. Content analysis was run by inductively drawing themes based on the elicited information from participants.

### Ethical considerations

The protocol for the study was approved by the relevant ethics committees of ministries of health in Ebonyi and Kano states, Nigeria. Before the interviews (both FGDs and IDIs), the participants were given information sheets/forms and the purpose of the study was explained to them before commencement of the study. Although the research team did not meet any minor during the interviews, the team planned to approach the husbands or caregivers of the minors to obtain consent while maintaining confidentiality.

## Results

### Demographic characteristics of the participants

The demographics showed that the women with fistula who participated in this study is presented in the Table 1 below.

**Table 1:** Demographics Characteristics of the Participants

Characteristics	FGD; total number of participants (N = 65)		IDIs (N = 44)	
	Ebonyi	Katsina	Ebonyi	Katsina
Interview category				
Women with women (at NOFIC)	12	12	9	9
Married men	10	11		
Married women	11	9		
Healthcare providers			10	10
Caregivers			3	3
Age category				
20–30 yrs	17	18	6	9
31–40 yrs	10	11	7	10
41–50 yrs	5	4	4	8
Average length of time that women lived with fistula (yrs)	6.5	6	6	6

### Awareness about causes and symptoms of obstetric fistula

#### Perceptions of women with fistula

There was lack of knowledge of fistula onset and its causes among women with fistula across northern and southeastern Nigeria, although knowledge was higher among participants in southeastern region. Some women lived with fistula for a long time but did not know what it was or what to do. Women rarely perceived the cause of fistula to be prolonged or obstructed labor, the most common cause of obstetric fistula. Instead, patients attributed the onset of urine leakage to consumption of high-sugar carbonated drinks (e.g. “La Casera”), lifting heavy loads, and coughing.

*“How it was happening to me then was that when I want to lift heavy chair or laugh, it will come out (urine) or during a cough or when I drink sugary things like mineral or La Casera, my whole cloth will soak.”* (FGD, repaired women, Ebonyi)

In both locations, some women blamed hospitals (and those attending to them) for causing their fistula following childbirth even in scenarios where they experienced extensive delay and prolonged labor at home before reaching the hospital. In recounting prolonged labor as a factor leading up to the development of their fistula, women often described waiting 48 h to seek emergency obstetric care.

*"This hospital may have caused this thing for me, maybe they did not return something into me properly, I am not thinking of something else in my heart.”* (IDI, Repaired woman, Kano)

“*My own is that it is my first pregnancy that resulted in the fistula. I had prolonged labor in Imo state, when they wanted to do C/S the baby came out. I labored all through in the hospital and they (hospital) gave me to cut and it resulted to fistula"* (FGD, Repaired women, Ebonyi)

Some women reported several unsuccessful surgeries attempts by health providers at private and secondary public facilities before they were referred to the NOFIC. Findings further indicated attribution of childbirth-related procedures to fistula cause. Some women think that when urine catheters are inserted into them during treatment, this leads them to develop a fistula.

*“The doctor carried catheter, say two and put inside me, three days later I called a nurse and said remove this thing I will carry my leg and enter the bathroom to ease myself. I said that I won’t have any problem.”* (IDI, Repaired Fistula woman, Ebonyi)

Awareness of the causes of the fistula was more common among women who have undergone repairs or other surgeries and who probably were counseled or referred during the treatment.

*“My experience is that I was small when women are still being circumcised. I sustained an injury in the bladder. I was taken to FMC when we got there a nurse directed us to come here to NOFIC for specialist care. It was done successfully...”* (FGD, repaired women, Ebonyi)

Some women, including those who delivered at home, described their fistula to be caused by witchcraft, saying that their landladies bewitched them, thereby indicating disharmony in the neighborhood.

*"Aunty I will say it is the devil because in my case I have lost three children and they were all males, you see the witches that are behind all these just wanted to take my life but God forbid”* (IDI, repaired fistula woman, Ebonyi).

These perceptions were also amplified by prophecies advocated by some church leaders who tell female members that they should never deliver children by cesarean section; this leads to women prolonging unattended labor while waiting for normal vaginal delivery.

#### Perceptions of community women

Awareness about fistula was higher among community married women than men and among participants in Ebonyi compared to Kano. However, knowledge of the causes of fistula was generally quite low. Some community women were aware that fistula is a result of problems during delivery, including the mismanagement of labor by uncertified doctors.

*"VVF (vesicovaginal fistula) is a problem that woman come across, maybe during and after delivery, maybe for example as I mentioned earlier on that when they take you to all those native doctors…. maybe they have already injured you inside your womb, then after that, you may have such problem*…” (FGD community women, Ebonyi)

Some of the community women interviewed identified that non-use of antenatal (ANC) services probably contributed to the development of fistula as most women do not go for ANC and husbands do not help their partners to receive appropriate care during pregnancy.

*“Some people have this habit, some will say they normally deliver at home, they won’t come to the hospital, you can find that sometimes among the women and sometimes from the men, and they will not bring their wives to the hospital…”* (FGD community women, Kano)

#### Community men’s perceptions

Community men’s lack of awareness of fistula is complicated by the fact that women who are living with fistula do not share their experiences with anyone—families, neighbors, and other community members,—primarily due to shame and lack of confidentiality. Some community men also think women avoid seeing doctors to avoid having to share their experiences living with fistula.

*“Any person who has suffered such ailment, will not like to disclose it to anybody. You know such sickness people will like to hide it because of the condition. For us, there is no way we can know who has suffered from such…”* (FGD community men, Ebonyi)

*“They (fistula clients) don’t inform their wards or their children. If they have this problem, they should bring it to the hospital” …”* (FGD community men, Kano)

Mixed views were reflecting an awareness of the fistula. Some community men knew about fistula only after a relative has experienced it, or after seeing an affected woman. None of the community men correctly identified the causes and symptoms of fistula and knew where to seek treatment. Some men perceived that access to fistula screening and treatment may be limited by geographic locations which also prevents women from accessing quality and emergency obstetric care.

*"There is no nearby hospital or PHC, the problem is numerous, where they go to obtain a card (ANC). It is only in Abakaliki, where some women go for antenatal… it is far from this community and also the place of delivery is far” (FGD Community Men, Ebonyi)*

Although knowledge about the cause of fistula was generally poor among men, some men thought that fistula could be prevented through timely use of ANC services. Other men shared concerns that women do not access ANC and timely delivery services due to transport challenges. Some men attributed the cause of fistula to the size of the woman's womb which may complicate labor and delivery.

*“The problem will occur because there is no mobility for taking the women to the hospital. If labor starts at night, she will visit the TBA for any assistance but in the process, she loses the baby and her life too” (FGD, Community Men, Ebonyi)*

“*Their womb is not big enough to carry a pregnancy, but God permitted them to be married and get pregnant. But when it is time for delivery, there must be a problem” (FGD, Community Men, Kano).*

#### Caregivers’ perceptions

Similar to community perspectives, quite a few of the caregivers had never heard about fistula before their relative developed it and sought treatment. Caregivers broadly described being unaware of the onset of fistula and its causes but elaborated on a range of perceived causes, including the physical position of a woman while giving birth and cesarean section.

*“…this ailment is when a girl squats to deliver and this delivery is not forthcoming…another case, this labor can cause the womb to press down on the bladder and this is when they get this problem…”* (IDI Female Caregiver, Kano)

Although some of the caregivers had never heard about fistula before their relative getting it, others attributed the cause of fistula to holding back urine for a long time.

“*Hajia, for us we have never seen it before, we just saw it, nobody has ever suffered it in my community. I swear for me, I have never heard, only on her did I know it” …. (IDI, female caregiver family member, Kano).*

*“Her own they said that the child stayed long, that is why this thing (fistula) occurred as people tell us. They say the problem is from the doctor. He allowed the girl to suffer long and that was why”* (IDI, female caregiver/family member, Ebonyi).

Some caregivers showed a complete lack of awareness about the causes and onset of fistula. Awareness of fistula improved after they accompanied their relatives during fistula repair. Others were still not clear about the causes of fistula even when providing support to a relative, probably because caregivers may not have benefited from counseling sessions preceding repairs.

“*I don’t know the meaning of the sickness; it’s just that my sister had the problem after C/S operation. I have seen people that are leaking urine but I don’t know the name of the sickness*” (IDI, female caregiver/family member, Ebonyi).

### Awareness about where to access care for fistula

Evidence from the FGDs and IDIs among all participants (women with fistula, community women, community men, and caregivers) showed lack of knowledge of where to get care for fistula, which led to inappropriate care-seeking, as well as women living with fistula for many years.

#### Women with fistula

Interviews with women indicated that knowledge of where to access care only improved with experience of fistula repair. Before arriving at the NOFIC on referral, many women had sought several alternative sources of care, including prayer houses and other private hospitals; some even resorted to self-medication. Women who had their fistulas repaired were eager to refer community members suffering from the same condition.

*“In my community, there is a young lady who is much older than me, she gave birth to her first child and had this problem. This lady isolates herself from the people and stays alone. I have promised myself that once I leave this hospital I will go and look for this lady because she lives very far in the village. I will tell her about this place, even if she will not have children at least let her live a normal and happy life.”* (IDI, repaired woman, Ebonyi).

“*It was in the night that I told him, he had to call a doctor who asked me to come to be examined after which the doctor said frankly this urination should be referred to the hospital*” (IDI, Repaired Fistula woman, Kano)

Even when women become aware of their condition, they still do not know where to access care except if someone else with experience or knowledge of receiving care refers the patient to a specific facility.

*“When I had this problem, I never took it seriously, I thought it was something else until I heard somebody say that another woman had the same problem, she came to the hospital and was attended to and she is well"* (IDI, repaired woman, Ebonyi).

#### Caregivers

Caregivers lacked knowledge of a repair center.

“*This thing happened 6 years ago, we didn’t go to any other place until July this year she came and complained that the pain from her private part became more severe*” (*IDI, Female Caregiver, Ebonyi).*

Lack of awareness of where to access care for fistula also led to people traveling long distances to seek treatment.

*“The problem disgraces a person. When a person gets that problem, she cannot go amid people. For three months that my sister gets this problem, she cannot go to church, she will stay in the home, be crying all the time. She even went to Abuja for treatment before they referred us to this place.” (IDI, Female caregiver, Ebonyi).*

*"When I went back and asked one woman that works in the hospital, she said frankly she does not know but she will inquire on our behalf but that she has never heard that it's treated in Kano” (IDI, Female Caregiver, Kano)*

Caregivers perceived that awareness must be created widely—nationwide—given that many people may not be aware of the NOFICs in geopolitical zones.

*I think lack of sensitization is the major one because so many people are not aware of this place especially the Aba area. I have never heard of this place and never knew this kind of place exists and I know it's like that for most people*” (*IDI, female caregiver, Ebonyi).*

#### Community women

Some community men, women, and caregivers did know where treatment for fistula could be accessed, though since it is less socially normative for women whose fistulas were repaired to not disclose their experience and care-seeking pathway, overall awareness remains low. This problem is compounded in some areas by the belief that fistula is evil imposed on them either by the gods or their neighbors.

*“Some don’t know, it’s even people that let me say from an urban area that would just explain to them that; this kind sickness, you go to the hospital and you will be cured. They go to the chemist and buy small drugs, if they give it to that person maybe they will say it’s somebody that is doing this to them.” (FGD community women, Ebonyi).*

### General care seeking behavior

Home delivery was common in both regions; some of the women with fistula had most of their babies at home or on the way to the hospital without any skilled care or even the help of an experienced neighbor. Women found it difficult to accept the consequences of their care-seeking behaviors that may have led to the development of fistula. While some women blame their health providers, others suspected neighbors and other relatives of evil thoughts. In some cases, women had up to eight deliveries at home and this trend was consistent in all locations.

*"It is the devil because you see those my eight pregnancies, nobody assisted me during delivery once I feel labor, I will just go to the backyard and take a warm shower and before the next 20–30 mins the baby will be out”* (IDI repaired woman, Ebonyi)

*"From the beginning when she went into labor, she spent two days and they did not inform me. It was in the morning of the second day that they came to tell me. When I was told in the morning, I went there, she was in it, so I said she should be taken to the hospital, they said they wanted to deliver at home”* (IDI repaired woman, Kano).

However, patients’ reported experiences at private secondary facilities indicate that there is also a gross lack of awareness on how to manage fistula among providers outside the NOFICs. This might be linked to why there were reportedly several attempts of 'trial and error' to repair women of their fistula with failed attempts as well as not correctly defining the causal root of the fistula to patients.

“*According to the doctor, he invited me to explain it to me. He asked me how old I was when I got married which I told him, so he said it was as a result of early marriage and early sex. He also asked me how I had my first sex and I told him that it was my husband that de-virgin me and he now said that it was early marriage because that was at 17 years".* (FGD Repaired woman Ebonyi).

#### NOFIC providers’ insights

Responses show high levels of knowledge of how to repair a fistula. Providers at NOFIC were aware that women seek care at different lower-level facilities or where there are no skilled providers trained in fistula repair. Many women had undergone several repair attempts by untrained or inexperienced providers before presenting to the NOFIC. Inexperienced providers may also actually complicate the woman’s fistula condition.

*"Yea, most of them visit various places and eventually come down with a mutilated kind of fistula and that's equally difficult. It is just that they (providers at other facilities) don't have the very important knowledge. Apart from being a medical doctor, you need to undergo training for this type of surgery, it is not just surgery you go and read on text book and come and practice, you have to see it done” ….(Fistula Surgeon, Ebonyi).*

*“Yes, you see being an Obgyn does not make a fistula surgeon. That has to be understood” (Fistula Surgeon, Kano)*

Medical social workers at NOFIC counsel the patients on arrival and admission before bookings. There seems to be appropriate patient counseling and education.

*"So maybe out of fear of exposure, they may not adhere to medical instructions. They come in at that point and help them relax and be able to accept instructions…” (IDI Medical Social Workers, Ebonyi)*

*“The first person to be in contact with, she will come here, and I will give the lecture. The lecture enlightens them about all they need to know about fistula and the reason of having fistula, why it occurs and how they come about it and how they find their way down to the hospital the source that gave them the information" (Theatre Nurse, Kano)*

NOFIC providers feel that women delay for years because of a lack of awareness of where to find treatment.

*“I think most patients don’t present early because of the awareness. Some come with a long history of leakage over several years. (Fistula Surgeon, Ebonyi).*

NOFIC providers expressed surprise that, despite their community mobilization efforts to increase awareness for fistula care services available in the area, community men and women still report poor awareness of the availability of fistula services. Providers report that they have conducted meetings at churches, and women’s groups, house-to-house, and market outreaches in selected communities. Providers also reported that awareness creation strategies and sources of information differed across locations. Media announcements e.g. radio was stronger in northern Nigeria (Kano) than in the southeast (Ebonyi) which was predominantly through interpersonal communication.

Providers believe that several women with fistula are still “hidden” and their family members do not know that treatment is available.

*“I remember last year… I led a team I combed the whole of Nsukka in Enugu state, I moved from market to market from church to church, met with community leaders. We moved from house to house for sensitization (Fistula Surgeon, Ebonyi).*

*Some of them say: “we heard it from the radio and friends”. They are calling people with fistula to come to the hospital for repairs and so on. Some of them will say they heard it from their friend” (Pre-operative Nurse, Kano)*

## Discussion

In this study, we investigated community perceptions about fistula causes and symptoms. We also sought to understand women's care-seeking pathways and health provider insights. Although the average length of years the women had lived with obstetric fistula was 6 years, none of the women was an adolescent at the time of the study. However, this does not negate the fact that some of these women may have developed obstetric fistula earlier in the adolescent age. Efforts must be made to include causes of fistula and other emergency obstetric conditions as parts of sex education modules at all level of education systems in Nigeria to raise obstetric fistula awareness.

Findings reveal poor awareness of fistula causes and symptoms and where to seek fistula repair, among men, women, and caregivers. Community members’ poor awareness of fistula onset and availability of fistula repair services in our study areas is consonant with other studies that highlight community misconceptions around the cause of fistula [16, 21, 22]. Given recent counseling and repair service experience, repaired fistula patients were more aware of the causes of fistula compared to men and other caregivers. Differences in awareness between women in this study likely reflect varied exposure to fistula-related information and treatment experiences in other private hospitals or referral points in women’s pathways to specialized fistula centers.

Geographic variation in awareness of and perceptions about fistula onset, cause, and availability of repair services was limited in this study. The only geographic variation was related to nomenclature (naming) of the cause of the condition due to language differences.

This study found that lack of awareness of fistula onset and where to get care for fistula led to inappropriate treatment seeking behavior. Poor awareness about where to seek care for fistula may also be reflective of women’s history of limited care-seeking for labor and delivery as well as lack of knowledge of the availability of health facilities and services in general [5, 23]. For example, many of the women interviewed did not seek facility-based care for childbirth. Institutional childbirth is often seen as expensive and unnecessary and women do not see the need to seek childbirth care, especially when other female members and the women themselves have had children born at home without any reported or notified complications [19]. Lack of women's autonomy and decision-making also present as barriers to seeking care [6]. This too has been reported in other countries [24–27].

Male partners’ poor awareness of causes and signs and symptoms of fistula contribute to preventing women from seeking timely care [28, 29]. In this study, men report that women may tend to hide reproductive health information from significant others. However, male partners are likely to influence women’s decisions to seek prompt obstetric care. Evidence regarding contributions or impact of male partner involvement in obstetric care seeking has not been widely published in Nigeria but has been described elsewhere [28–30]. Women may also delay delivery care-seeking as late as possible to reduce the number of admission days and waiting hours before delivery.

However, care-seeking for fistula remains challenging for women [30–35]. Some of the women with fistula sought treatment in varying places including TBAs, herbalists, and doctors in private facilities with no experience of fistula symptoms and repairs.

Providers at NOFIC were very knowledgeable about fistula onset and treatment, as expected, given the centers' specialized nature. If fistula awareness campaigns such as mass media are championed by the NOFIC to provide the link between the lower cadre providers and communities, more women may be mobilized for repairs. However, the NOFIC must redesign their case-finding and referral strategies to ensure adequate information diffusion about fistula, its causes and available repair services.

Our study describes the context of barriers that intersect with women’s and communities’ awareness around fistula onset, occurrence, cause, and service availability in Nigeria. Awareness creation among lower-level providers must include early detection, basic counseling, and referral mechanisms at lower cadre health facilities as well as educating lower-level CHEWs, nurses, and TBAs on their limits to the delivery of obstetric care [26]. Any design of community interventions should incorporate prevention—including quality ANC, encouraging institutional delivery, and identifying obstetric emergency conditions both at home and the lower-level facilities, and knowing how to refer to the NOFIC. It is also necessary to identify community platforms that engage and target women to increase fistula awareness and dispel the misconceptions in rural villages [15, 16]. Women who were successfully repaired can serve as change agents or “ambassadors” to improve awareness about fistula in rural communities in Nigeria.

A multi-pronged intervention approach is required to increase awareness of the causes of fistula, particularly the dangers of giving birth at home and not delaying seeking care if labor is prolonged [36, 37]. Although media can yield a wider geographic coverage, it may not be as effective as in-person contact. Despite stipulations of the national strategic framework for the elimination of obstetric fistula in Nigeria highlights some health systems and behavior change communication strategies to improve health provider performance including catheterization, family planning, and emergency obstetric and neonatal care, partner and community leader’s involvement awareness of fistula onset and where to access care remains low, necessitating a divergent approach at the community level [15].

This study has some limitations. First, it did not explore the interaction of other barriers to fistula care services such as financial, psycho-social, cultural, and opportunity cost barriers. Second, the sample for the study was small. Therefore, quantitative studies of larger samples of fistula patients may usefully examine the relative contributions of different barriers to fistula care, contextualizing low awareness in comparison to other challenges. Additional research is needed on how different interventions can reduce access barriers and improve access to fistula repair.

## Conclusions

Research findings reveal a very lack of awareness of obstetric fistula onset and awareness of the availability of repair services at the community level and among lower-level health service providers. There is a need to explore multi-pronged strategies for increasing awareness of obstetric fistula and where treatment services are available among women and other community members. There is a need to create awareness among providers in lower capacity facilities on how to screen for fistula and where to refer clients for treatment.

## Data Availability

All data (transcripts) generated in this study are available and shall be provided upon request.

## Abbreviations

SSA: Sub-Saharan Africa
NOFiCs: National Obstetric Fistula Centres
PHC: Primary health care
ANC: Antenatal care
FGDs: Focus group discussion
IDIs: In-dept interviews
LGAs: Local Government Areas
CHEWs: Community Health Extension Workers
TBAs: Traditional Birth Attendants
US: United States
IRB: Institutional Review Board
NHREC: National Health Research Ethics Committee
FMOH: Federal Ministry of Health
